# Repeats of Unusual Size in Plant Mitochondrial Genomes: Identification, Incidence and Evolution

**DOI:** 10.1534/g3.118.200948

**Published:** 2018-12-18

**Authors:** Emily L. Wynn, Alan C. Christensen

**Affiliations:** School of Biological Sciences, University of Nebraska, Lincoln, Nebraska 68588-0666

**Keywords:** plant mitochondrial genomes, repeated sequence, genome rearrangement, organelle genome evolution

## Abstract

Plant mitochondrial genomes have excessive size relative to coding capacity, a low mutation rate in genes and a high rearrangement rate. They also have abundant non-tandem repeats often including pairs of large repeats which cause isomerization of the genome by recombination, and numerous repeats of up to several hundred base pairs that recombine only when the genome is stressed by DNA damaging agents or mutations in DNA repair pathway genes. Early work on mitochondrial genomes led to the suggestion that repeats in the size range from several hundred to a few thousand base pair are underrepresented. The repeats themselves are not well-conserved between species, and are not always annotated in mitochondrial sequence assemblies. We systematically identified and compared these repeats, which are important clues to mechanisms of DNA maintenance in mitochondria. We developed a tool to find and curate non-tandem repeats larger than 50bp and analyzed the complete mitochondrial sequences from 157 plant species. We observed an interesting difference between taxa: the repeats are larger and more frequent in the vascular plants. Analysis of closely related species also shows that plant mitochondrial genomes evolve in dramatic bursts of breakage and rejoining, complete with DNA sequence gain and loss. We suggest an adaptive explanation for the existence of the repeats and their evolution.

It has long been known that plant mitochondrial genomes are much larger than those of animals (Ward, B. L. *et al.* 1981) and include significant amounts of non-coding DNA (Schuster, W. and A. Brennicke 1994). These genomes also often have repeats of several kb, leading to multiple isomeric forms of the genome (Folkerts, O. and M. R. Hanson 1989; Klein, M. *et al.* 1994; Palmer, J. D. and L. A. Herbon 1988; Palmer, J. D. and C. R. Shields 1984; Siculella, L. *et al.* 2001; Sloan, D. B. *et al.* 2010; Stern, D. B. and J. D. Palmer 1986). Plant mitochondrial genomes have very low mutation rates, but paradoxically have such high rearrangement rates that there is virtually no conservation of synteny (Drouin, G. *et al.* 2008; Palmer, J. D. and L. A. Herbon 1988; Richardson, A. O. *et al.* 2013; Wolfe, K. *et al.* 1987).

In addition to the large, frequently recombining repeats, there are often other repeated sequences in the size range of 1kb and lower (Arrieta-Montiel, M. P. *et al.* 2009; Forner, J. *et al.* 2005). Ectopic recombination between these non-tandem repeats has been shown to increase when double-strand breakage is increased, or in plants mutant for DNA maintenance genes (Abdelnoor, R. V. *et al.* 2003; Shedge, V. *et al.* 2007; Wallet, C. *et al.* 2015). Understanding the repeats is critical to understanding the mechanisms of DNA maintenance and evolution in plant mitochondria, yet they have never been systematically identified and analyzed. In addition to being infrequently and inconsistently annotated and described in mitochondrial genome sequences, repeats are often described as long, short and intermediate-length (Arrieta-Montiel, M. P. *et al.* 2009; Davila, J. I. *et al.* 2011; Miller-Messmer, M. *et al.* 2012). The repeats are sometimes thought to be distributed into two size classes (one of up to several hundred bp and another of several kb), but this is derived from early studies of Arabidopsis and a few other species in which repeats were described and annotated (Alverson, A. J. *et al.* 2011b; Andre, C. *et al.* 1992; Arrieta-Montiel, M. P. *et al.* 2009; Davila, J. I. *et al.* 2011; Folkerts, O. and M. R. Hanson 1989; Sugiyama, Y. *et al.* 2005).

The most likely hypothesis that explains the peculiar characteristics of plant mitochondrial genomes is that double-strand break repair (DSBR) is abundantly used in plant mitochondria, perhaps to the exclusion of nucleotide excision and mismatch repair pathways (Christensen, A. C. 2014; Christensen, A. C. 2018). Double-strand break repair is very accurate when the repair is template-based, accounting for the low mutation rate in genes, but the nonhomologous end-joining or break-induced-replication pathways can account for the creation of repeats and chimeric genes, expansions, and loss of synteny through rearrangements.

The inconsistent reporting and annotation of repeated sequences leads to a number of questions. What is the best way to discover and characterize them? Is the size distribution really bimodal in angiosperms? Are there repeats in the mitochondria of other groups of green plants? How do they differ between groups? Can they be followed through evolutionary lineages like genes? Are the repeats themselves somehow adaptive, or are they a side-effect of DSBR that is neutral or nearly neutral? The availability in recent years of complete mitochondrial genome sequences across a wide variety of taxa of green plants allows us to begin addressing these questions. We describe a computational strategy for finding non-tandem repeats within plant mitochondrial genomes. Using this tool we describe the phylogenetic distribution of repeats in both size classes, examine their evolution in a family of closely related angiosperms, and propose an hypothesis for the evolutionary significance of the repeats and the DSBR processes that produce them.

## Materials and Methods

### Sequence data and manipulation

Table 1 lists the mitochondrial genome sequences that were downloaded as FASTA format files from GenBank (https://www.ncbi.nlm.nih.gov/genbank/). BLAST searches (Altschul, S. F. *et al.* 1990) were done using version 2.7.1 on a Linux-based machine. In addition to the sequences shown in Table 1, mitochondrial genomes from several *Brassica* species were used to compare close relatives. These sequences are as follows: *Brassica carinata*; JF920287, *Brassica rapa*; JF920285, *Brassica oleracea fujiwase*; AP012988, *Brassica napus polima*; FR715249, *Brassica juncea*; JF920288. Alignments were done using the clustalW implementation in the VectorNTI 11.5 software package (ThermoFisher).

**Table 1:** List of species and mitochondrial DNA accession numbers

Species	Group	Subgroup	Accession
Auxenochlorella protothecoides	Chlorophytes	Chlorophyta	KC843974.1
Botryococcus braunii	Chlorophytes	Chlorophyta	LT545992.1
Bracteacoccus aerius	Chlorophytes	Chlorophyta	KJ806265.1
Chlamydomonas eustigma	Chlorophytes	Chlorophyta	BEGY01000520.1
Chlamydomonas reinhardtii	Chlorophytes	Chlorophyta	EU306617.1
Chlorella sp. ArM0029B	Chlorophytes	Chlorophyta	KF554428.1
Dunaliella salina strain GN	Chlorophytes	Chlorophyta	KX641169.1
Eudorina sp.	Chlorophytes	Chlorophyta	KY442294.1
Gloeotilopsis planctonica	Chlorophytes	Chlorophyta	KX306823.1
Gloeotilopsis sarcinoidea	Chlorophytes	Chlorophyta	KX306822.1
Hariotina sp. MMOGRB0030F	Chlorophytes	Chlorophyta	KU145405.1
Kirchneriella aperta	Chlorophytes	Chlorophyta	NC_024759.1
Lobosphaera incisa	Chlorophytes	Chlorophyta	KP902678.1
Microspora stagnorum	Chlorophytes	Chlorophyta	KF060942.1
Mychonastes homosphaera	Chlorophytes	Chlorophyta	NC_024760.1
Neochloris aquatica	Chlorophytes	Chlorophyta	NC_024761.1
Nephroselmis olivacea	Chlorophytes	Nephroselmidophyceae	AF110138.1
Oltmannsiellopsis viridis	Chlorophytes	Chlorophyta	DQ365900.1
Ourococcus multisporus	Chlorophytes	Chlorophyta	NC_024762.1
Polytoma uvella	Chlorophytes	Chlorophyta	NC_026572.1
Prototheca wickerhamii	Chlorophytes	Chlorophyta	U02970.1
Pseudendoclonium akinetum	Chlorophytes	Chlorophyta	AY359242.1
Tetradesmus obliquus	Chlorophytes	Chlorophyta	CM007918.1
Trebouxiophyceae sp. MX-AZ01	Chlorophytes	Chlorophyta	JX315601.1
Ulva flexuosa	Chlorophytes	Chlorophyta	KX455878.1
Ulva linza	Chlorophytes	Chlorophyta	NC_029701.1
Bathycoccus prasinos	Chlorophytes	Prasinophytes	NC_023273.1
Cymbomonas tetramitiformis	Chlorophytes	Prasinophytes	KX013548.1
Micromonas sp. RCC299	Chlorophytes	Prasinophytes	FJ859351.1
Monomastix sp. OKE-1	Chlorophytes	Prasinophytes	KF060939.1
Ostreococcus tauri	Chlorophytes	Prasinophytes	CR954200.2
Prasinoderma coloniale	Chlorophytes	Prasinophytes	KF387569.1
Pycnococcus provasolii	Chlorophytes	Prasinophytes	GQ497137.1
Pyramimonas parkeae	Chlorophytes	Prasinophytes	KX013547.1
Anthoceros angustus*	Anthocerotophyta	Anthocerotophyta	NC_037476.1
Leiosporoceros dussii*	Anthocerotophyta	Anthocerotophyta	NC_039751.1
Megaceros aenigmaticus	Anthocerotophyta	Anthocerotophyta	EU660574.1
Phaeoceros laevis	Anthocerotophyta	Anthocerotophyta	GQ376531.1
Aneura pinguis	Marchantiophyta	Marchantiophyta	KP728938.1
Calypogeia neogaea*	Marchantiophyta	Marchantiophyta	NC_035980.1
Marchantia polymorpha	Marchantiophyta	Marchantiophyta	NC_001660.1
Pleurozia purpurea	Marchantiophyta	Marchantiophyta	NC_013444.1
Treubia lacunosa	Marchantiophyta	Marchantiophyta	JF973315.1
Tritomaria quinquedentata*	Marchantiophyta	Marchantiophyta	NC_037041.1
Anomodon attenuatus	Bryophytes	Bryophytes	JX402749.1
Atrichum angustatum	Bryophytes	Bryophytes	KC784956.1
Bartramia pomiformis	Bryophytes	Bryophytes	KC784955.1
Brachythecium rivulare	Bryophytes	Bryophytes	KR732319.1
Bucklandiella orthotrichacea	Bryophytes	Bryophytes	KP742835.1
Buxbaumia aphylla	Bryophytes	Bryophytes	KC784954.1
Climacium americanum	Bryophytes	Bryophytes	KC784950.1
Codriophorus aciculare	Bryophytes	Bryophytes	KP453983.1
Funaria hygrometrica	Bryophytes	Bryophytes	KC784959.1
Hypnum imponens	Bryophytes	Bryophytes	KC784951.1
Nyholmiella gymnostoma	Bryophytes	Bryophytes	KX578030.1
Nyholmiella obtusifolia*	Bryophytes	Bryophytes	NC_031767.1
Orthotrichum callistomum	Bryophytes	Bryophytes	KX578029.1
Orthotrichum speciosum*	Bryophytes	Bryophytes	NC_026121.1
Oxystegus tenuirostris	Bryophytes	Bryophytes	KT326816.1
Physcomitrella patens	Bryophytes	Bryophytes	NC_007945.1
Ptychomnion cygnisetum	Bryophytes	Bryophytes	KC784949.1
Racomitrium elongatum*	Bryophytes	Bryophytes	NC_026890.1
Sanionia uncinata	Bryophytes	Bryophytes	KP984757.1
Sphagnum palustre	Bryophytes	Bryophytes	KC784957.1
Stoneobryum bunyaense	Bryophytes	Bryophytes	KX578031.1
Syntrichia filaris	Bryophytes	Bryophytes	KP984758.1
Tetraphis pellucida	Bryophytes	Bryophytes	KC784953.1
Tetraplodon fuegianus	Bryophytes	Bryophytes	KT373818.1
Ulota phyllantha	Bryophytes	Bryophytes	KX578033.1
Zygodon viridissimus	Bryophytes	Bryophytes	KX711975.1
Chaetosphaeridium globosum	Streptophyta	Charophyta	AF494279.1
Chara vulgaris	Streptophyta	Charophyta	AY267353.1
Chlorokybus atmophyticus	Streptophyta	Charophyta	NC_009630.1
Closterium baillyanum	Streptophyta	Charophyta	NC_022860.1
Entransia fimbriata	Streptophyta	Charophyta	KF060941.1
Klebsormidium flaccidum	Streptophyta	Charophyta	KP165386.1
Nitella hyalina	Streptophyta	Charophyta	JF810595.1
Roya obtusa	Streptophyta	Charophyta	KF060943.1
Ophioglossum californicum	Tracheophyta	Fern	NC_030900.1
Psilotum nudum	Tracheophyta	Fern	NC_030952.1
Huperzia squarrosa	Tracheophyta	Lycophyte	NC_017755.1
Selaginella moellendorffii	Tracheophyta	Lycophyte	GL377739.1
Cycas taitungensis	Tracheophyta	Gymnosperm	AP009381.1
Ginkgo biloba	Tracheophyta	Gymnosperm	KM672373.1
Welwitschia mirabilis	Tracheophyta	Gymnosperm	NC_029130.1
Aegilops speltoides	Tracheophyta	Angiosperm	AP013107.1
Ajuga reptans	Tracheophyta	Angiosperm	KF709392.1
Allium cepa	Tracheophyta	Angiosperm	KU318712.1
Ammopiptanthus mongolicus*	Tracheophyta	Angiosperm	NC_039660.1
*Arabidopsis thaliana*	Tracheophyta	Angiosperm	BK010421
Arabis alpina*	Tracheophyta	Angiosperm	NC_037070.1
Batis maritima	Tracheophyta	Angiosperm	KJ820684.1
Beta vulgaris	Tracheophyta	Angiosperm	BA000024.1
Betula pendula	Tracheophyta	Angiosperm	LT855379.1
Boea hygrometrica	Tracheophyta	Angiosperm	JN107812.1
Bombax ceiba*	Tracheophyta	Angiosperm	NC_038052.1
Brassica nigra	Tracheophyta	Angiosperm	KP030753.1
Bupleurum falcatum*	Tracheophyta	Angiosperm	NC_035962.1
Butomus umbellatus	Tracheophyta	Angiosperm	KC208619.1
Cannabis sativa	Tracheophyta	Angiosperm	KU310670.1
Capsicum annuum	Tracheophyta	Angiosperm	KJ865410.1
Carica papaya	Tracheophyta	Angiosperm	EU431224.1
Castilleja paramensis	Tracheophyta	Angiosperm	KT959112.1
Chrysanthemum boreale*	Tracheophyta	Angiosperm	NC_039757.1
Citrullus lanatus	Tracheophyta	Angiosperm	GQ856147.1
Citrus sinensis*	Tracheophyta	Angiosperm	MG736621.1
Cocos nucifera	Tracheophyta	Angiosperm	KX028885.1
Corchorus capsularis	Tracheophyta	Angiosperm	KT894204.1
Daucus carota subsp. sativus	Tracheophyta	Angiosperm	JQ248574.1
Diplostephium hartwegii	Tracheophyta	Angiosperm	KX063855.1
Eruca vesicaria	Tracheophyta	Angiosperm	KF442616.1
Geranium maderense	Tracheophyta	Angiosperm	NC_027000.1
Glycine max	Tracheophyta	Angiosperm	JX463295.1
Gossypium hirsutum	Tracheophyta	Angiosperm	NC_027406.1
Helianthus annuus	Tracheophyta	Angiosperm	CM007908.1
Heuchera parviflora	Tracheophyta	Angiosperm	KR559021.1
Hibiscus cannabinus	Tracheophyta	Angiosperm	MF163174.1
Hordeum vulgare	Tracheophyta	Angiosperm	AP017300.1
Hyoscyamus niger	Tracheophyta	Angiosperm	KM207685.1
Ipomoea nil	Tracheophyta	Angiosperm	AP017303.1
Lagerstroemia indica	Tracheophyta	Angiosperm	KX641464.1
Leucaena trichandra*	Tracheophyta	Angiosperm	NC_039738.1
Liriodendron tulipifera	Tracheophyta	Angiosperm	NC_021152.1
Lolium perenne	Tracheophyta	Angiosperm	JX999996.1
Lotus japonicus	Tracheophyta	Angiosperm	JN872551.2
Medicago truncatula	Tracheophyta	Angiosperm	KT971339.1
Millettia pinnata	Tracheophyta	Angiosperm	JN872550.1
Mimulus guttatus	Tracheophyta	Angiosperm	JN098455.1
Nelumbo nucifera*	Tracheophyta	Angiosperm	KR610474.1
Nicotiana tabacum	Tracheophyta	Angiosperm	BA000042.1
Nymphaea colorata*	Tracheophyta	Angiosperm	KY889142.1
Oryza sativa Indica	Tracheophyta	Angiosperm	NC_007886.1
Phoenix dactylifera	Tracheophyta	Angiosperm	JN375330.1
Platycodon grandiflorus*	Tracheophyta	Angiosperm	NC_035958.1
Populus tremula	Tracheophyta	Angiosperm	KT337313.1
Raphanus sativus	Tracheophyta	Angiosperm	NC_018551.1
Rhazya stricta	Tracheophyta	Angiosperm	KJ485850.1
Ricinus communis	Tracheophyta	Angiosperm	HQ874649.1
Rosa chinensis*	Tracheophyta	Angiosperm	CM009589.1
Salix suchowensis	Tracheophyta	Angiosperm	KU056812.1
Salvia miltiorrhiza	Tracheophyta	Angiosperm	KF177345.1
Schrenkiella parvula	Tracheophyta	Angiosperm	KT988071.2
Senna tora*	Tracheophyta	Angiosperm	NC_038053.1
Silene latifolia	Tracheophyta	Angiosperm	HM562727.1
Sinapis arvensis	Tracheophyta	Angiosperm	KM851044.1
Sorghum bicolor	Tracheophyta	Angiosperm	DQ984518.1
Spinacia oleracea	Tracheophyta	Angiosperm	KY768855.1
Styphnolobium japonicum*	Tracheophyta	Angiosperm	NC_039596.1
Tripsacum dactyloides	Tracheophyta	Angiosperm	NC_008362.1
Triticum aestivum	Tracheophyta	Angiosperm	AP008982.1
Utricularia reniformis*	Tracheophyta	Angiosperm	NC_034982.1
Vicia faba	Tracheophyta	Angiosperm	KC189947.1
Vigna angularis	Tracheophyta	Angiosperm	AP012599.1
Viscum album	Tracheophyta	Angiosperm	NC_029039.1
Vitis vinifera complete	Tracheophyta	Angiosperm	FM179380.1
*Zea mays* strain NB	Tracheophyta	Angiosperm	AY506529.1
Ziziphus jujuba	Tracheophyta	Angiosperm	KU187967.1

### Repeat Analysis

Custom Python scripts are in Supplementary Materials. The script ROUSFinder.py (Supplemental File S1) uses blastn to perform a pairwise ungapped comparison of a sequence with itself, both strands separately, using a word size of 50, E value of 10,000, reward for a match +1, penalty for a mismatch -20. The script then concatenates the two output files and the full length self-identity is deleted. Alignments are then sorted and compared to identify and remove duplicate repeats, and an output file containing each distinct repeat in fasta format is created. The sorting by size allows the script to automate the curation of the repeats by comparing the query start and end coordinates of each identified repeated sequence with the subject start and end coordinates of repeated sequences of the same size. When there are more than two copies of a repeat, BLAST does not report every pairwise hit, so the output file of FASTA-formatted sequences of repeats is then used as a query with the entire genome as subject to locate every copy of that repeat, create a table, and a table of binned sizes. The output can also be formatted for GenBank annotation. MultipleRepeats.py (Supplemental File S2) automates running ROUSFinder.py on every sequence within a directory.

Prior work identifying repeats, especially in *Arabidopsis thaliana* (Arrieta-Montiel, M. P. *et al.* 2009) showed that although BLAST is very useful, it has some characteristics that make it difficult to automate curation of the identified matches of non-tandem DNA sequences. For example, if there are a number of mismatches in a repeat, sometimes BLAST will identify subsets of the repeat sequence, or not give the same alignments when two imperfect repeats are used as queries of the entire genome. When there are three or more copies of a repeat, BLAST will also not identify every possible pairwise alignment, giving a subset instead. When examining the repeats in a single species, these problems can be solved by additional manual curation and inspection of the species. However, for automated curation of sequences from multiple species, some compromises have to be made. The simplest way to curate the repeated sequences is to ensure that the sizes of each repeat in a pair are the same. This ensures that repeats can be matched with each other by examining the coordinates of each copy to find all copies of the repeated sequence. In *Arabidopsis thaliana*, an ungapped blastn search with a match reward of +1 and a mismatch penalty of -18 or lower ensured that different copies of the repeats were the same length, allowing automated curation. We therefore set the penalty parameter to -20 to make the automated curation reliable and fast. In order to more carefully examine the repeats in a single species, the script ROUSFinder2.py (Supplemental File S3) allows the user to set the match reward and mismatch penalty parameters on the command line.

After an initial analysis of sequences available in early 2018, we added additional species to the data in late 2018. These additional species are indicated in Table 1 by an asterisk. These include two hornworts, two liverworts, 3 bryophytes and 14 angiosperms. These new species do not change the patterns or conclusions compared to the earlier analysis, providing additional validation of the use of BLAST and the curation methods described.

### Data Availability

The authors state that all data necessary for confirming the conclusions presented in this article are represented fully within the article, including python scripts in Supplemental Material and accession numbers of DNA sequences shown in Table 1. Supplemental material available at Figshare: https://doi.org/10.25387/g3.7425680.

## Results

### Repeats in plant mitochondrial genomes

The existence of large non-tandem repeats in plant mitochondrial genomes is well known by now, but they have not been systematically identified and analyzed. Prior studies used variations of BLAST (Altschul, S. F. *et al.* 1990) to find repeats (Alverson, A. J. *et al.* 2011a; Alverson, A. J. *et al.* 2010; Alverson, A. J. *et al.* 2011b; Liu, Y. *et al.* 2014) or REPuter (Hecht, J. *et al.* 2011; Kurtz, S. and C. Schleiermacher 1999). Other available software packages specifically identify tandem repeats, or repeats matching known repetitive sequences. Due to the ready availability of BLAST and the flexibility of its use, and because most prior work used it, we wrote and used a Python script called ROUSFinder.py that uses BLAST to identify non-tandem repeats within mitochondrial genomes. The parameters for identification of a sequence repeat were relatively stringent and included a blastn word size of 50, and match/mismatch scores of +1/-20. Any choice of parameters will necessarily identify some false positives and false negatives. These parameters were chosen in order to find duplicate copies of sequence that were either recently created or recently corrected by gene conversion. As described in the Methods, they were also chosen to enable automated curation of the repeats that are found in the first iteration of BLAST. A duplication longer than 100 bases that has several mismatches or a gap in the center of the repeat unit will be identified as two different repeats by this script. However, mismatches in the center of one copy of a repeat are indicative of either two independent events producing the two parts of the repeat, or mutation and drift that have escaped gene conversion. Because we are concerned with the recombination behavior of the repeats we therefore chose to call these two different repeats. To analyze and identify repeats in a single sequence for further study or annotation would require additional manual curation of the output. The word size parameter of 50 is chosen to make the output more manageable. Reducing the word size identifies numerous smaller repeats, but the smaller the repeats get, the more complex the curation task of distinguishing identical sequences from similar ones of the same size – a task that at this point still needs to be done manually. Reducing the word size does not change the conclusions about any of the potentially recombinogenic repeats that also distinguish the major groups in the plant kingdom. Previous work in *Arabidopsis thaliana* has shown that crossover products between non-tandem repeats of 556bp or smaller in accession Col-0 and 204bp or smaller in accession Ws are undetectable by PCR (Wallet, C. *et al.* 2015), similar to prior results using Southern blots (Arrieta-Montiel, M. P. *et al.* 2009; Davila, J. I. *et al.* 2011; Sakamoto, W. *et al.* 1996)

The species we used represent a significant subset of the complete mitochondrial genome sequences from green plants in GenBank and are shown in Table 1. Sequences available on GenBank are not a random sample across taxa (food crops are very over-represented, for example), so to reduce sampling bias somewhat we used only one species per genus. Incomplete sequences or sequences with gaps or wildcard characters (such as N, R, Y, etc.) are not handled well by BLAST without further curation, so these were not used. Species with multiple distinct chromosomes were also not used because of the additional layer of complexity from inter- and intra-chromosomal repeats. The full output is in Supplemental Table S1. The repeats seen in plant mitochondrial genomes are much larger than those found in random sequence (data not shown), suggesting that they arise from specific biological processes and are not stochastic.

### Phylogenetic clustering

**Figure 1:**
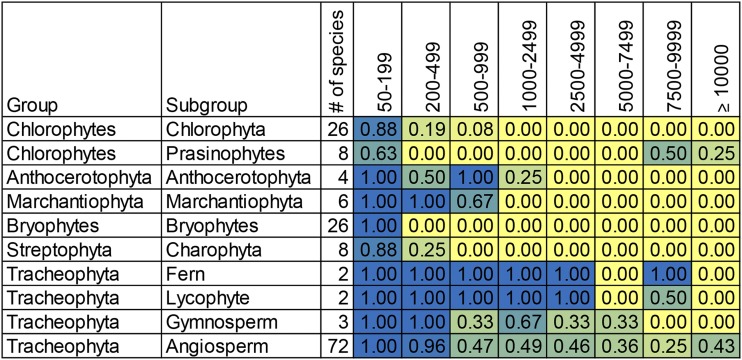
Size distributions of repeats in groups of species. The number of species represented in each group is shown. Headings indicate the bins of repeat sizes and the numbers indicate the fraction of species in that group that have at least one repeat of that size. Heat map color coding is blue for one and yellow for zero.

The distribution of repeat sizes forms distinct clusters between broad phylogenetic groups (see Figure 1). Because there are different numbers of species in each group, and some species have an order of magnitude more total repeats than others, we represent the data as the fraction of species within that group that have at least one repeat within a given size range. The complete output is in Supplemental Table S1. Within the chlorophytes, repeats of greater than 200bp are rare. The exceptions are the prasinophytes (discussed below) and a few interesting cases. *Chlamydomonas reinhardtii* has a 532 bp inverted repeat at the termini of its linear chromosome. *Dunaliella salina*, *Kirchneriella aperta* and *Polytoma uvella* have novel structures at a small number of loci that consist of overlapping and nested repeats and palindromes (Smith, D. R. *et al.* 2010). The function of these structures is unknown, but they are unusual and not common in the chlorophytes. The prasinophyte group resembles the rest of the chlorophytes in having no non-tandem repeats greater than 200bp but many of them include two copies of a single very large repeat between 9.5 and 14.4 kb. This is similar to many chloroplast genomes and it is possible that this structure is involved in replication (Bendich, A. J. 2004). The bryophytes generally resemble the chlorophytes; there are no repeats longer than 200bp.

In contrast to the chlorophytes and bryophytes, the Marchantiophyta (liverworts) and Anthocerotophyta (hornworts) have repeats greater than 200bp in size, but none bigger than 1131bp. The other lineages of streptophytic green algae (referred to as charophytes in GenBank) resemble the chlorophytes albeit with a slightly higher upper limit. In this group the largest repeat is found in *Chlorokybus atmophyticus* and is 291bp.

The ferns and lycophytes are strikingly different from the previous groups. Unfortunately the number of species sequenced is low. They have large numbers of repeats and the repeat sizes range well above 200bp, up to 10 kb. Some members of these groups, such as *Huperzia*, are similar to the bryophytes, but others are large and have significant repeat content (Guo, W. *et al.* 2017). These groups are underrepresented among available mitochondrial sequences, in part due to the complexity caused by the repetitive nature of lycophyte and fern mitochondrial genomes (Grewe, F. *et al.* 2009), but the patterns are noticeably different from the nonvascular plants described above.

The angiosperms are represented very well in the sequence databases. Only one member of this group does not have any repeats larger than 200bp (*Medicago truncatula*). A small number of angiosperms lack repeats larger than 1 kbp, and approximately half include repeats larger than 9 kbp. *Silene conica*, a species with multiple large chromosomes not included in our dataset has a nearly 75kb sequence found in both chromosomes 11 and 12 (Sloan, D. B. *et al.* 2012). Gymnosperms are also underrepresented, but appear to be similar to the other vascular plants. Interestingly, the gymnosperms *Ginkgo biloba* and *Welwitschia mirabilis* resemble angiosperms, while *Cycas taitungensis* is more similar to ferns. The *C. taitungensis* mitochondrion has numerous repeats, including many that are tandemly repeated. Five percent of this genome consists of the mobile Bpu element, a remarkable level of repetitiveness (Chaw, S. M. *et al.* 2008).

It is only in the vascular plants that the number and size of repeated sequences in mitochondrial genomes has been expanded. The vascular plants generally only have mitochondrial genomes a few times larger than the bryophytes, liverworts and hornworts, but the repeats are expanded well beyond proportionality to size. Some taxa, such as the Geraniaceae, Plantago, and *Silene* include species with significantly expanded mitochondrial DNA (Park, S. *et al.* 2015; Parkinson, C. L. *et al.* 2005; Sloan, D. B. *et al.* 2012). These species are outliers in the mitochondrial genome sizes and the number of repeats, but the underlying DNA replication, recombination and repair processes are likely to be the same. There appears to have been a significant change in mitochondrial DNA maintenance mechanisms coincident with the origin of the vascular plants.

### Repeat sizes and frequency in angiosperms

**Figure 2:**
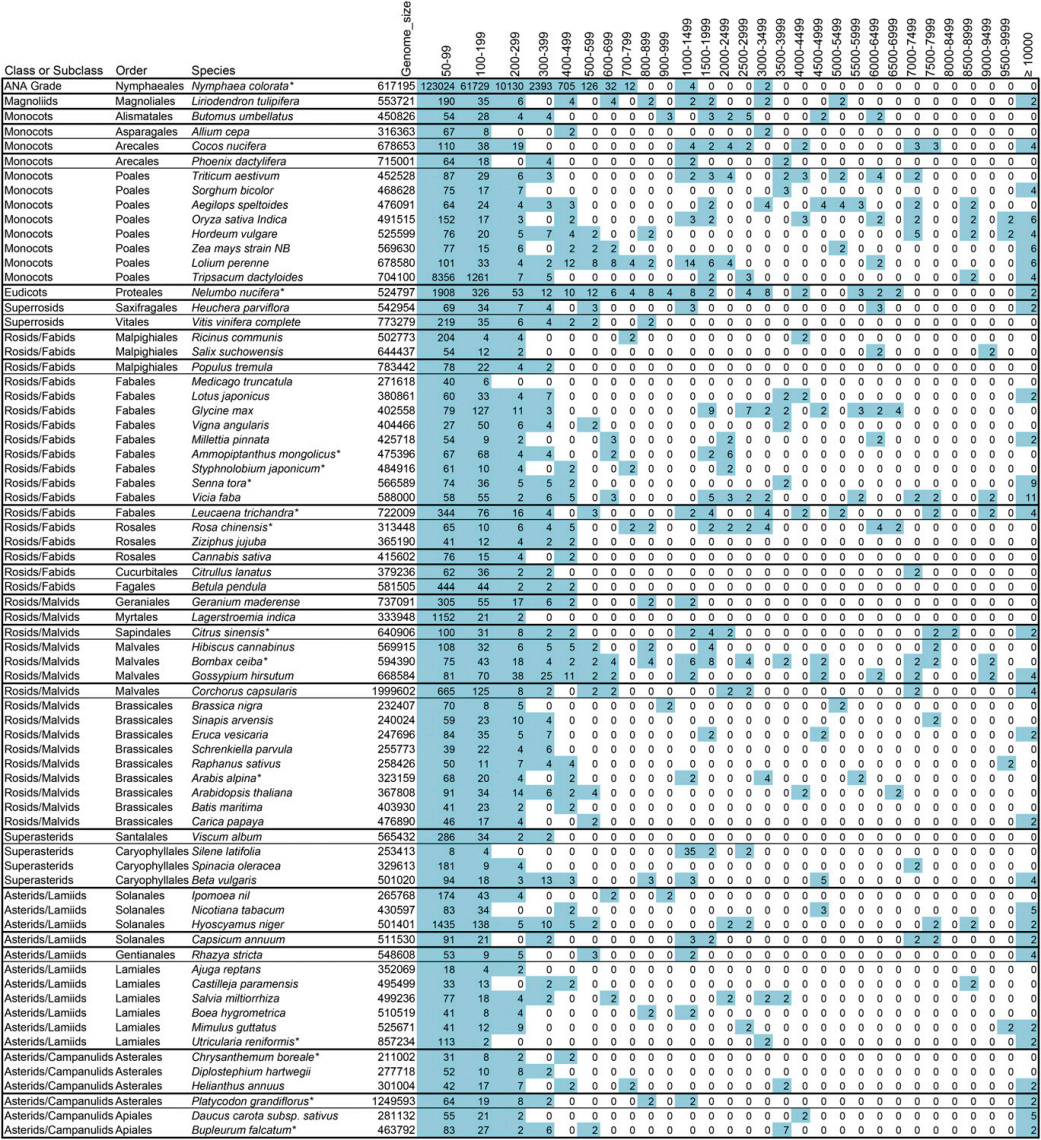
Distribution of repeat sizes among angiosperms. Species are sorted by the phylogenetic groups as described by the Angiosperm phylogeny group (Cole *et al.* 2017). The number of repeats of each size class is shown. Blue shading indicates a number greater than zero.

Large repeats of several kilobases have been identified in several species and shown to be recombinationally active, isomerizing angiosperm mitochondrial genomes (Folkerts, O. and M. R. Hanson 1989; Klein, M. *et al.* 1994; Palmer, J. D. and L. A. Herbon 1988; Palmer, J. D. and C. R. Shields 1984; Siculella, L. *et al.* 2001; Sloan, D. B. 2013; Stern, D. B. and J. D. Palmer 1986). A few species have been reported to lack such structures (Palmer, J. D. 1988). The first comprehensive catalog of repeated sequences shorter than 1000 base pairs was done in *Arabidopsis thaliana*, and they were shown to be recombinationally active in some mutant backgrounds, but not generally in wild type (Arrieta-Montiel, M. P. *et al.* 2009; Davila, J. I. *et al.* 2011; Miller-Messmer, M. *et al.* 2012; Shedge, V. *et al.* 2007). Is the spectrum of repeat sizes in Arabidopsis, and its bimodality, typical for angiosperms? Figure 2 illustrates the presence of repeats in the size range of 50bp to over 10,000 bp in 72 angiosperms, sorted by the class and order of the species. While individual species often have a bimodal distribution of sizes, there is no size range that is universally absent from the distribution. Thirteen of the 72 species have no repeats larger than 600bp, leaving open the question of whether those particular mitochondrial genomes isomerize through recombination. All of the other species have a large repeat of somewhere between 600bp and 65kbp. There is no pattern of repeat size distribution or total size with the phylogenetic group or total mitochondrial genome size, suggesting that these are not produced by stochastic processes, and suggesting that they occur and change faster than speciation does.

### Alignment of repeats within the Brassicales

In order to test the hypothesis that the repeated sequences change rapidly compared to speciation events, leading to the lack of pattern in the Angiosperm orders, we analyzed 6 closely related species in the Brassica genus. Within the *Brassica* genus there are three diploid species: *Brassica rapa*, *Brassica nigra* and *Brassica oleracea*, and three allotetraploid species (Cheng, F. *et al.* 2017). The diploid nuclear genomes are called the A, B and C genomes, respectively. Based on both nuclear and mitochondrial sequences it appears that *Brassica carinata* has the *B. nigra* and *B. oleracea* nuclear genomes (BBCC) and the *B. nigra* mitochondrial genome, while *Brassica juncea* has the *B. nigra* and *B. rapa* nuclear genomes (BBCC) and the *B. rapa* mitochondrial genome. *Brassica napus* has two subspecies, *polima* and *napus*. Both have the *B. oleracea* and *B. rapa* nuclear genomes (AACC), but *B. napus polima* appears to have the *B. rapa* mitochondrial genome and *B. napus napus* has the *B. oleracea* mitochondrial genome (Chang, S. *et al.* 2011; Franzke, A. *et al.* 2011; Grewe, F. *et al.* 2014). Thus it appears that the hybridization event between *B. oleracea* and *B. rapa* occurred at least twice, with each species being the maternal parent. In the analysis below we use the *B. napus polima* mitochondrial genome. We compared these Brassica species to *Raphanus sativus* and *Sinapis arvensis* as outgroups. These species are the closest relatives of the Brassicas with complete mitochondrial genome sequences (Grewe, F. *et al.* 2014). Several of these species were mapped prior to genomic sequencing, and repeated sequences and mitochondrial genome isomerization was observed (Palmer, J. D. 1988; Palmer, J. D. and L. A. Herbon 1986).

**Figure 3:**
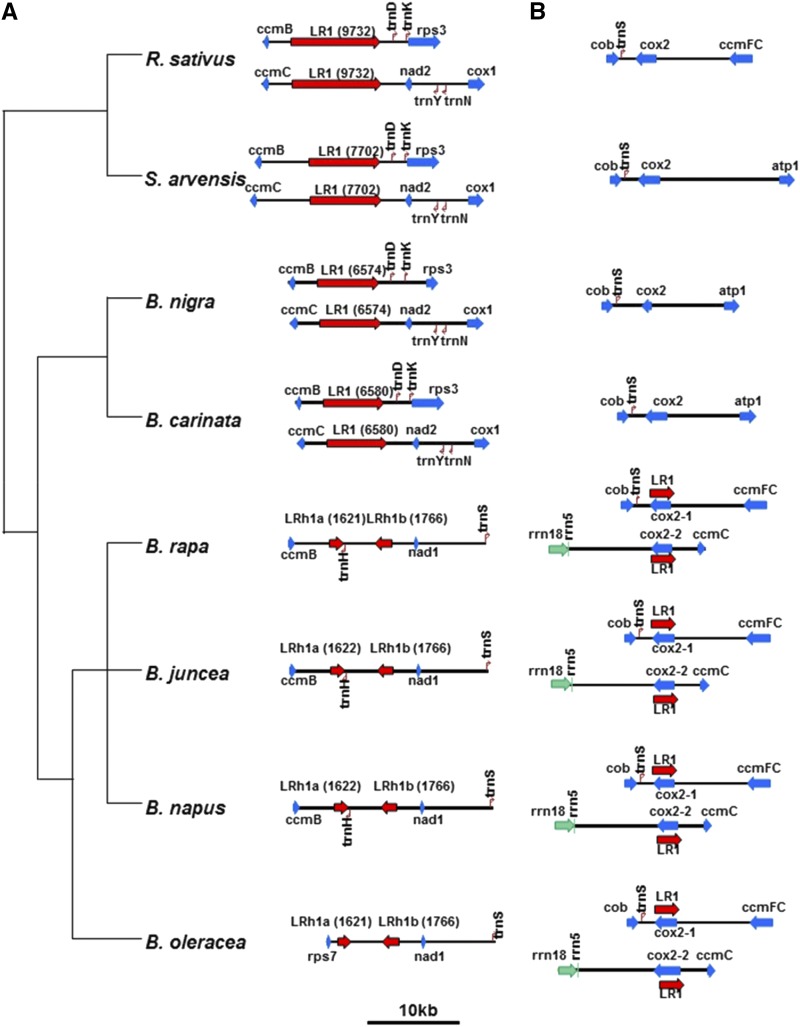
Alignment of long repeats in the Brassicales. A phylogenetic tree is shown at left, derived from Grewe *et al.* (Grewe, F. *et al.* 2014). Part A aligns the longest repeat in Group A (*R. sativus*, *S. arvensis*, *B. nigra* and *B. carinata*) and shows the genes flanking them. The homologous single-copy sequence from *B. rapa*, *B. napus*, *B. juncea* and *B. oleracea* is also shown. Part B aligns the longest repeat in Group B (*B. rapa*, *B. napus*, *B. juncea* and *B. oleracea*), and shows the homologous single-copy region in Group A. Red arrows indicate the long repeats that were used to align all sequences in the two parts of the figure. Blue indicates genes in the flanking regions that may or may not be conserved or rearranged. Green indicates rRNA genes and small arrows represent tRNA genes. Branch lengths in the tree are not to scale. The sequences are depicted at the scale shown in the figure.

All eight of these species include one pair of long repeats, ranging in length from 1.9kb to 9.7kb. However, these species show an interesting pattern. *B. nigra*, *B. carinata*, *R. sativus* and *S. arvensis*, hereafter referred to as group A, each have two copies of a 6.5 to 9.7kb repeat that is only present as single copy sequence in the mitochondria of *B. rapa*, *B. oleracea*, *B. napus* and *B. juncea*, herafter referred to as group B (see Figure 3). The group B species each have two copies of a long repeat 1.9kb long that is present as single-copy sequence in group A. Figure 3 shows these repeated sequences, aligned only to each other and placed onto the known phylogenetic tree of the Brassicales (Grewe, F. *et al.* 2014). The longest repeats are aligned, and the genes flanking them are shown. Part A shows the long repeat and neighboring sequences from the A group and the homologous single-copy sequences from the B group. Part B compares the long repeat from the B group to the single-copy homologous region from the A group.

Grewe *et al.* examined the synonymous substitution rates in genes of Brassicales mitochondrial genomes (Grewe, F. *et al.* 2014) and found them to be very low, consistent with most land plants. However, the presence of repeats allows mutations in non-coding DNA to be examined qualitatively. The long repeats in the A group differ by large block substitutions and insertion/deletions (alignments are shown in Supplemental Figure S1). Where two copies are present in a species there are very few difference between copies, and they are generally near the boundaries of the repeats. Although significant differences can arise during speciation events, both copies of a repeat within a species remain identical. This supports the hypothesis that copies of repeated DNA are maintained as identical sequence by frequent recombination and gene conversion.

The long repeat of *B. nigra* and *B. carinata* underwent massive change in the lineage leading to the B group of Brassica species (see Figure 3). The first 1.6kb and the last 1.7kb of the long repeat in the A group are conserved in the B group, and the *ccmB* gene still flanks the repeat on one side. However, the last 1.7kb are inverted and separated from the first 1.6kb by 3.3kb of a sequence of unknown origin. An additional difference is seen in *B. oleracea* wherein *rps7* now flanks the repeat rather than *ccmB*. Other major changes appear to have occurred in the time since *B. nigra* diverged from the ancestor of *B. oleracea* and *B. rapa*; a comparison of the complete mitochondrial genomes of *B. rapa* and *B. nigra* reveal at least 13 segments of DNA that have been rearranged. No major rearrangements have occurred between *B. nigra* and *B. carinata*, nor between *B. rapa*, *B. juncea* and *B. napus polima*. *B. oleracea* differs from *B. rapa* by approximately six rearrangement events (Grewe, F. *et al.* 2014).

At the same time that the long repeat of the A group was being dramatically altered in the lineage leading to *B. rapa* and *B. oleracea*, a new long repeat appeared in the B group, which includes the coding sequence of the *cox2* gene. This new long repeat is maintained throughout this group of four species, and the flanking genes are also conserved (see Figure 3; sequence alignments are shown in Supplemental Figure S2). The *cox2* gene is single copy in the A group and is in a nearly syntenic arrangement with neighboring genes.

## Discussion

The availability of complete mitochondrial genome sequences from many taxa of green plants allows us to compare the abundance and size distribution of non-tandem repeats across taxa. Although such repeats have been known for some time, their functions (if any) and evolution are largely mysterious. It has been suggested that their existence and maintenance are outgrowths of double-strand break repair events such as nonhomologous end-joining (NHEJ), break-induced replication (BIR) and gene conversion (Christensen, A. C. 2018). We describe here a Python script that uses BLAST (Altschul, S. F. *et al.* 1990) to find non-tandem repeats within sequences, and use it to analyze plant mitochondrial DNA. In addition, comparison of repeats between closely related species within the Brassicales showed that repeat differences between species were largely due to rearrangements and block substitutions or insertions, which could be due to NHEJ and BIR, while the two copies of the repeat were identical within a species, suggesting continuing repair by gene conversion or homologous recombination.

Repeats in mitochondria appear to be more abundant and larger in the vascular plants than in the non-vascular taxa. This suggests that the first vascular common ancestor of lycophytes, ferns, gymnosperms and angiosperms acquired new mechanisms of mitochondrial genome replication and repair that led to a proliferation of repeats and increases in repeat size and mitochondrial genome size. Complete sequences of more species, particularly in the lycophytes and ferns, is necessary to add clarity but the ancestor of vascular plants evidently made a transition to increased use of double-strand break repair in their mitochondria, leading to the genomic gymnastics seen today.

The analysis of repeats in the *Brassica* species suggests that mitochondrial genomes can remain relatively static for long periods of time, but can also diverge very rapidly by rearrangements, sequence loss, and gain of sequences of unknown origin. This pattern resembles punctuated equilibrium (Gould, S. J. and N. Eldredge 1977). The mechanisms and frequency are unknown, but it suggests that a lineage can experience a burst of genome recombination, breakage and rejoining, dramatically rearranging and altering the mitochondrial genome, as if it had been shattered and rebuilt. These events occur on a time scale that is faster than that of speciation, leading to high levels of divergence, and loss of synteny.

Qualitative differences have been described between the repeats shorter and longer than about 1kb (Arrieta-Montiel, M. P. *et al.* 2009; Klein, M. *et al.* 1994; Mower, J. P. *et al.* 2012). In general the largest repeats within a species have been found to recombine constitutively, leading to isomerization of the genome into multiple major forms. The shortest repeats (less than 50bp) may be involved in homologous recombination events only rarely, while those of intermediate size, generally in the 100s of base pairs, can recombine in response to genome damage or in DNA maintenance mutants, but do not normally do so in unstressed, non-mutant plants, as noted above. The intermediate size repeats have been primarily analyzed in *Arabidopsis thaliana*, and have been found to recombine in abnormal conditions. In plants treated with ciprofloxacin (which induces mitochondrial double-strand breaks), or in mutants of the mitochondrial *recG* homolog, repeats of 452, 249, 204 and 126bp were seen to recombine (Wallet, C. *et al.* 2015). In mutants of *msh1* (which results in high levels of ectopic recombination), there was some recombination seen between repeats as small as 70bp, but none in repeats of 50bp or smaller (Davila, J. I. *et al.* 2011). This suggests a changing spectrum of function and activity correlated with size, which could also vary by species.

Functional analysis of repeat recombination can be done by analyzing clones big enough to include the repeats (Klein, M. *et al.* 1994), by long read sequencing (Shearman, J. R. *et al.* 2016), PCR (Wallet, C. *et al.* 2015) or by Southern blotting (Arrieta-Montiel, M. P. *et al.* 2009; Sakamoto, W. *et al.* 1996). Functional analysis of the large repeats is an important step in understanding plant mitochondrial genome structure and evolution (Guo, W. *et al.* 2016; Guo, W. *et al.* 2017; Sloan, D. B. 2013) and may reveal different patterns of recombination between species, which would reveal important differences in the replication and repair machinery and dynamics.

We doubt that there is an adaptive advantage to large size and abundant rearrangements in the genomes of plant mitochondria. We suggest that these are correlated traits accompanying the adaptive advantage of a greatly increased reliance on double-strand break repair. DNA repair is critically important because damage is more likely in mitochondria than the nucleus due to the presence of reactive oxygen species. In animals, the mitochondrial mutation rate is high, but the reduced mitochondrial genome size minimizes the number of potential mutational targets (Lynch, M. *et al.* 2006; Smith, D. R. 2016). However, with multiple copies of mitochondrial DNA in each cell, an alternative strategy in a high DNA damage environment is to increase the use of template DNA in repair. The accuracy of double-strand break repair when a template is used is accompanied by the creation of chimeras, rearrangements and duplications when templates are not identical or cannot be found by the repair enzymes. Dramatic expansions, rearrangements and losses, accompanied by low substitution rates in genes is characteristic of flowering plant mitochondria. Selection on gene function maintains the genes, while the expansions and rearrangements must be nearly neutral. Once mitochondria evolved very efficient double-strand break repair, and a mechanism for inducing double-strand breaks at the sites of many types of damage, more primitive mechanisms, such as nucleotide excision repair can and have been lost (Gualberto, J. M. *et al.* 2014; Gualberto, J. M. and K. J. Newton 2017) without obvious evolutionary cost.

The adaptive value of increased and efficient double-strand break repair is probably to avoid mutations in the essential genes of mitochondria, and is possible because of the abundance of double-stranded template molecules in each cell. However this mechanism of repair has an additional correlated trait. There are bacterial species, such as *Deinococcus radiodurans*, that excel at double-strand break repair and can rebuild even significantly fragmented genomes (Krisko, A. and M. Radman 2013) while also being able to minimize radiation-induced damage (Sharma, A. *et al.* 2017). While *D. radiodurans* is notoriously resistant to ionizing radiation, the adaptive value is thought to be desiccation resistance, because dehydration is more likely to have been experienced than extreme radiation in the history of the lineage, and also produces double-strand breaks (Mattimore, V. and J. R. Battista 1996). Radiation resistant bacteria in unrelated phylogenetic groups show more genome rearrangements and loss of synteny than their radiation sensitive relatives (Repar, J. *et al.* 2017), suggesting that abundant double-strand break repair is the cause of both the resistance to significant double-strand breakage and the loss of synteny. An interesting possibility is that very efficient double-strand break repair in plant mitochondria also confers desiccation resistance as a correlated trait. Because mitochondria are metabolically active immediately upon imbibition of seeds, DNA damage must be repaired very efficiently and rapidly (Paszkiewicz, G. *et al.* 2017). Efficient repair of desiccation-mediated damage in all cellular compartments is a prerequisite to being able to produce seeds or spores for reproduction. It is possible that the DNA repair strategy of plant mitochondria was one of several factors (including desiccation resistance of the nuclear and plastid genomes, presumably by distinct mechanisms) that are beneficial to vascular plants. The evidence of the repeats suggests that the transition to double-strand break repair in mitochondria occurred at approximately the same time as the transition to vascularity in plants, and it may have been one of several traits that enabled their success. In addition, once the life cycles of land plants included periods of desiccation in spores and seeds, double-strand breakage would have increased, accompanied by increases in rearrangements, expansions, and chimeras. The mechanisms of double-strand break repair continue to be important for understanding the evolution of plant mitochondrial genomes.
